# Protective effect of statins in patients with sepsis-associated encephalopathy: a retrospective cohort study

**DOI:** 10.3389/fneur.2026.1756916

**Published:** 2026-03-12

**Authors:** Tieqiao Zhou, Lei Li, Jiping Yi, Xiaoxiang Gong, Fengzhen Huang

**Affiliations:** 1 The First People’s Hospital of Chenzhou Affiliated to the University of South China, Chenzhou, Hunan, China; 2 The First Affiliated Hospital of Xiangnan University, Chenzhou, Hunan, China; 3 Department of Pediatrics, The Second Xiangya Hospital, Central South University, Changsha, Hunan, China

**Keywords:** MIMIC IV database, mortality, sepsis, sepsis-associated encephalopathy, statins

## Abstract

**Background:**

Sepsis-associated encephalopathy (SAE) is a severe neurological complication of sepsis. Statins may exerted protective effects in sepsis and its complications by reducing the dysregulated inflammatory response. However, it remains unknown whether statins provide any protective effect on SAE.

**Methods:**

The data for this study were extracted from the MIMIC-IV database. Cox proportional hazards regression models were constructed to assess the association between statin therapy and mortality rate for in-hospital, 30-day, 90-day, 180-day, and 365-day. Kaplan–Meier survival curves were used to estimate survival probabilities between the non-statin group and statin group. Subgroup analysis was conducted to investigate potential variations in the effects of statin treatment on clinical outcomes among different groups.

**Results:**

A total of 4,707 patients with SAE were included in the study, with 2,387 in the non-statin group and 2,320 in the statin group. The findings indicated that the use of statins was linked to a considerable decrease in mortality rates. Patients who were administered statins experienced lower in-hospital mortality and demonstrated enhanced survival rates at 30, 90, 180, and 365 days when compared to those not receiving statins. Further analysis of atorvastatin showed that the subgroup exhibited a similarly consistent reduction in mortality across all time points in comparison to the non-statin group. Interestingly, the protective effect associated with statin use remained significant regardless of the statin type, dosage, or exposure time.

**Conclusion:**

The use of statins was associated with short-term and long-term mortality among SAE patients admitted to the ICU. This association was observed irrespective of statin type, dosage, or exposure time. It is necessary to conduct large-scale prospective studies to further explore the relationship between statins and the prognosis of patients with SAE.

## Background

Sepsis-associated encephalopathy (SAE) is a severe neurological complication arising from sepsis, characterized by acute brain impairment that occurs as a secondary consequence of systemic infection, without direct central nervous system infection (1, 2). The incidence of SAE is particularly high among individuals with sepsis, with prevalence rates reaching up to 70% (3). The pathophysiology of SAE remained poorly understood, and studies suggest that it may involve mechanisms such as diffuse neuroinflammation, disruption of the blood–brain barrier, and immune dysregulation (4–6). Patients with SAE exhibit a broad spectrum of neurological symptoms, varying from subtle cognitive impairments to severe delirium and even profound coma, with symptoms like agitation and dysautonomia occurring less commonly (1). Diagnosing SAE is challenging, as it requires to exclude the direct central nervous system infection or other types of encephalopathy. The mortality rate associated with SAE is significantly higher than that of patients with sepsis, and a considerable number of survivors enduring long-term cognitive deficiencies and persistent neurological impairments (7). Therefore, early identification and prompt intervention are crucial to improve patient outcomes. Nevertheless, there are still no effective therapeutic strategies for this challenging condition so far.

Statins are inhibitors of 3-hydroxy-3-methylglutaryl-coenzyme A reductase, and are extensively used in cardiovascular and cerebrovascular diseases due to its cholesterol-lowering properties. Recently, statins have gained increasing attention for their pleiotropic properties (7). Studies indicated that statins may exerted protective effects in sepsis and its complications by mitigating the dysregulated inflammatory response. Yu et al. showed that statin administration notably decreased short-term mortality and shorten the duration of mechanical ventilation in critically ill patients (8). Zheng et al. reported that low-dose statins remarkably lowered all-cause mortality rates at 30 days, 90 days, and 1 year in critically ill patients with heart failure (9). Mao et al. discovered that statins could lead to shorter ICU stays and lower mortality rates at 30 days and 90 days in patients suffering from Acute Respiratory Distress Syndrome (ARDS) (10). However, it remains unknown whether statins provide any protective effect against SAE.

In this study, our real-world study from the MIMIC-IV database aims to evaluate the potential efficacy of statin treatment in patients with SAE. In addition, we also investigate the impact of atrovastatin, statin dosage, and statin exposure time on clinical outcomes, with the goal of offering insightful guidance into optimal therapeutic strategies for this vulnerable population.

## Methods

### Data source and study population

The data for this study were extracted from the MIMIC-IV database [version 3.1, (11, 12)], a comprehensive electronic health record repository that included de-identified clinical information from patients between 2008 and 2022. Fengzhen Huang has successfully obtained the access certificate for the database (Record IDs: 63858817 and 63858818) after completing the required examination. The database utilization was approved by the Institutional Review Boards of the Massachusetts Institute of Technology and Beth Israel Deaconess Medical Center.

Sepsis was defined as a suspected infection accompanied by an acute rise in the Sequential Organ Failure Assessment (SOFA) score ≥ 2 (13). Delirium was an acute condition characterized by fluctuations in attention and consciousness (14). Sepsis-associated encephalopathy (SAE) was diagnosed in septic patients who had a Glasgow Coma Scale (GCS) score <15 or presented with delirium (3). We recruited patients with SAE who were admitted to the ICU for the first time, had an ICU stay duration ≥24 h, and aged ≥18 years. Patients with exclusion criteria: (1) primary brain injury, such as cerebrovascular disease, meningitis or encephalitis, brain tumor, epilepsy, traumatic brain injury; (2) neurodegenerative diseases, mental disorders, or with alcohol or drug abuse; (3) metabolic encephalopathy, hepatic encephalopathy, hypertensive encephalopathy, and other kidney or liver diseases affecting consciousness; (4) severe electrolyte imbalances (Sodium < 120 mmol/L or >150 mmol/L) or glycemic disturbances (Glucose < 54 mg/dL or >180 mg/dL).

### Clinical variables

The extracted demographic information included age, weight, gender, race, marital status, and insurance status. The laboratory parameters included white blood cell (WBC), platelet count, hemoglobin, potassium, sodium, glucose, creatinine, blood urea nitrogen (Bun), aniongap, bicarbonate, partial thromboplastin time (PTT), and international normalized ratio (INR). The vital signs included mean peripheral oxygen saturation (SpO_2_), mean heart rate, mean temperature, mean respiration rate, mean systolic blood pressure (SBP), and mean diastolic blood pressure (DBP). Additionally, urine output data was recorded. The disease severity scores included sepsis-related organ failure assessment (SOFA) score, Glasgow coma scale (GCS) score, acute physiology score III (APSIII) score, and oxford acute severity of illness (OASIS) score. The therapy included the use of aspirin, vasoactive agent, and ventilation. The comorbidities included hypertension, diabetes, hyperlipidemia, cardiovascular disease, peripheral Vascular Disease (PVD), positive culture results, and Charlson comorbidity index (CCI). The process of data extraction was conducted by Structured Query Language (SQL).

### Outcomes

Patients were divided into two groups: the non-statin group and the statin group. Statin exposure was defined as the receipt of at least one dose of any statins during their hospital stay. In addition, SAE patients were subdivided into specific groups based on their use of atorvastatin, the dosage of statins, and the exposure time of statins. The primary clinical outcome was mortality rate for in-hospital, 30-day, 90-day, 180-day, and 365-day. The impact of statin therapy on these outcomes was evaluated to determine its potential role in improving survivals among SAE patients. Subgroup analyses was conducted to investigate potential variations in the effects of statin treatment on clinical outcomes among different groups.

### Statistical analysis

Statistical analyses were performed using R software (v4.4.0). The proportion of missing data for all indicators was less than 20%, and the missing values were randomly imputed with the “mice” package in R. Continuous variables were compared using the Mann–Whitney U test, while categorical variables were analyzed using Chi-square tests. Propensity score matching (PSM) was used to balance the baseline characteristics by nearest neighbor matching and 1:1 ratio (15). Cox proportional hazards regression models were constructed to assess the association between statin therapy and mortality rate, and the hazard ratio (HR) with 95% confidence interval (CI) was calculated. Kaplan–Meier survival curves were used to estimate survival probabilities across different groups, analyzed by the log-rank test. All statistical tests were two-sided, and a *p*-value < 0.05 was considered statistically significant.

## Results

### Baseline characteristics

**Figure 1:**
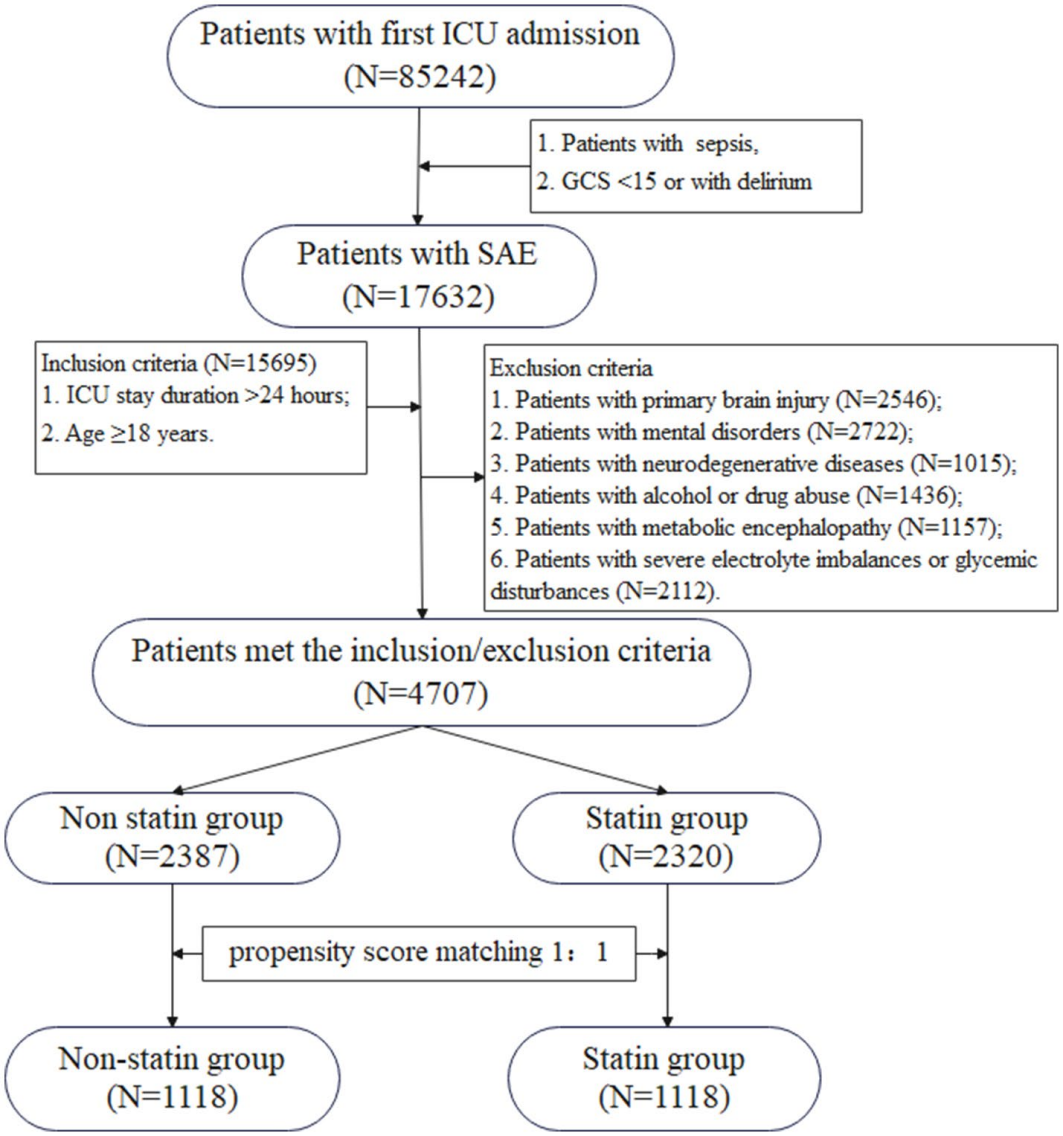
The process of SAE patients selection. ICU, intensive care unit; CS, Glasgow Coma Scale; SAE, sepsis-associated encephalopathy.

The patient selection process was presented in Figure 1. We identified a total of 85,242 patients who were admitted to the ICU for the first time, with 17,632 diagnosed with SAE. After applying the inclusion and exclusion criteria, 4,707 patients were ultimately selected (Supplementary Table 1). Among these individuals, 2,387 did not receive statin therapy and were classified as the non-statin group, while 2,320 patients received statin treatment and were categorized as the statin group. As detailed in Table 1, the statin group consisted of older, heavier patients, with a higher male proportion, compared to the non-statin group. There was a greater number of patients in the statin group who were receiving aspirin and vasoactive agents. Furthermore, the statin group also had a higher incidence of comorbidities such as hypertension, diabetes, cardiovascular disease, and peripheral vascular disease (PVD), and accompanied by a higher CCI score. After propensity score matching (PSM), each group contained of 1,118 patients each, ensuring a well-matched comparison. The standardized mean differences (SMD) for all variables were < 0.1 and *p*-values > 0.05.

**Table 1:** Baseline characteristics of SAE patients before and after PSM.

Variable	Before PSM					After PSM				
	Overall (4,707)	Non-statin (*n* = 2,387)	Statin (2,320)	*p*	SMD	Overall (2,236)	Non-statin (1,118)	Statin (1,118)	*p*	SMD
Demographics										
Age (median[IQR])	71.76 [60.64, 81.38]	66.33 [52.82, 79.40]	75.63 [67.05, 82.78]	<0.001	0.627	75.06 [64.67, 83.31]	75.64 [64.35, 84.61]	74.41 [64.99, 82.16]	0.031	0.054
Weight (median[IQR])	77.00 [64.50, 91.20]	75.00 [62.85, 90.75]	78.05 [66.27, 92.03]	<0.001	0.033	76.55 [64.00, 91.00]	75.50 [63.20, 90.00]	77.50 [64.90, 91.00]	0.055	0.018
Gender (%)										
Female	2,001 (42.5)	1,097 (46.0)	904 (39.0)	<0.001	0.142	973 (43.5)	481 (43.0)	492 (44.0)	0.670	0.020
Male	2,706 (57.5)	1,290 (54.0)	1,416 (61.0)			1,263 (56.5)	637 (57.0)	626 (56.0)		
Race (%)										
White	3,293 (70.0)	1,618 (67.8)	1,675 (72.2)	0.001	0.096	1,603 (71.7)	804 (71.9)	799 (71.5)	0.851	0.010
Other	1,414 (30.0)	769 (32.2)	645 (27.8)			633 (28.3)	314 (28.1)	319 (28.5)		
Marital status (%)										
Married	2,424 (51.5)	1,158 (48.5)	1,266 (54.6)	<0.001	0.121	1,170 (52.3)	591 (52.9)	579 (51.8)	0.641	0.021
Other	2,283 (48.5)	1,229 (51.5)	1,054 (45.4)			1,066 (47.7)	527 (47.1)	539 (48.2)		
Insurance (%)										
Medicare	3,042 (64.6)	1,301 (54.5)	1,741 (75.0)	<0.001	0.440	1,581 (70.7)	792 (70.8)	789 (70.6)	0.926	0.006
Other	1,665 (35.4)	1,086 (45.5)	579 (25.0)			655 (29.3)	326 (29.2)	329 (29.4)		
Laboratory index										
WBC (median[IQR])	9.80 [7.10, 14.20]	10.50 [7.10, 15.40]	9.40 [7.10, 13.20]	<0.001	0.149	10.10 [7.20, 14.40]	10.25 [6.93, 14.97]	10.00 [7.32, 13.90]	0.440	<0.001
Platelet (median[IQR])	204.00 [148.00, 274.50]	207.00 [143.00, 284.00]	201.00 [152.00, 264.00]	0.470	0.051	205.00 [149.00, 278.25]	205.00 [144.00, 281.00]	206.00 [155.25, 278.00]	0.387	0.012
Hemoglobin (median[IQR])	10.60 [9.00, 12.30]	10.50 [8.90, 12.20]	10.70 [9.10, 12.40]	0.003	0.102	10.50 [9.00, 12.10]	10.50 [9.10, 12.20]	10.60 [9.00, 12.10]	0.865	0.007
Potassium (median[IQR])	4.10 [3.70, 4.50]	4.10 [3.70, 4.50]	4.10 [3.80, 4.50]	<0.001	0.068	4.10 [3.70, 4.50]	4.10 [3.70, 4.50]	4.10 [3.80, 4.50]	0.219	0.017
Sodium (median[IQR])	139.00 [136.00, 141.00]	139.00 [136.00, 141.00]	139.00 [136.00, 141.00]	0.117	0.059	139.00 [136.00, 141.00]	139.00 [136.00, 141.00]	139.00 [136.00, 141.00]	0.677	0.009
Glucose (median[IQR])	116.00 [98.00, 138.00]	115.00 [97.00, 137.00]	117.00 [100.00, 140.00]	0.002	0.093	117.00 [98.00, 139.00]	117.00 [97.00, 140.00]	117.00 [100.00, 139.00]	0.594	0.019
Creatinine (median[IQR])	1.00 [0.80, 1.50]	0.90 [0.70, 1.40]	1.10 [0.80, 1.70]	<0.001	0.140	1.00 [0.80, 1.60]	1.00 [0.80, 1.50]	1.10 [0.80, 1.70]	0.121	0.037
Bun (median[IQR])	21.00 [14.00, 35.00]	20.00 [13.00, 34.00]	22.00 [16.00, 36.00]	<0.001	0.125	22.00 [16.00, 37.00]	22.00 [16.00, 37.00]	22.00 [15.00, 37.00]	0.887	0.014
Aniongap (median[IQR])	14.00 [12.00, 16.00]	14.00 [12.00, 16.00]	14.00 [12.00, 16.00]	0.580	0.031	14.00 [12.00, 16.00]	14.00 [12.00, 16.00]	14.00 [12.00, 16.00]	0.713	0.021
Bicarbonate (median[IQR])	24.00 [21.00, 27.00]	24.00 [21.00, 27.00]	25.00 [22.00, 28.00]	<0.001	0.193	24.00 [21.00, 27.00]	24.00 [21.00, 27.00]	24.00 [21.00, 28.00]	0.445	0.014
PTT (median[IQR])	31.10 [27.60, 36.90]	30.50 [27.30, 35.60]	31.70 [27.90, 38.20]	<0.001	0.146	31.10 [27.60, 36.50]	30.70 [27.70, 35.60]	31.40 [27.60, 37.08]	0.056	0.038
INR (median[IQR])	1.20 [1.10, 1.50]	1.30 [1.10, 1.50]	1.20 [1.10, 1.50]	0.068	0.018	1.30 [1.10, 1.50]	1.30 [1.10, 1.50]	1.30 [1.10, 1.58]	0.579	0.036
Vital signs										
SpO_2_ mean (median[IQR])	97.08 [95.70, 98.33]	96.97 [95.55, 98.33]	97.16 [95.84, 98.36]	0.013	0.089	96.91 [95.52, 98.22]	96.90 [95.50, 98.28]	96.93 [95.54, 98.16]	0.823	0.038
Heart rate mean (median[IQR])	85.28 [75.36, 96.79]	88.63 [77.31, 101.44]	82.54 [74.07, 91.92]	<0.001	0.367	84.42 [74.50, 96.14]	84.55 [74.59, 96.96]	84.40 [74.32, 95.69]	0.718	0.020
Temperature mean (median[IQR])	36.81 [36.56, 37.12]	36.85 [36.59, 37.19]	36.77 [36.54, 37.06]	<0.001	0.159	36.79 [36.54, 37.10]	36.80 [36.54, 37.09]	36.79 [36.54, 37.10]	0.817	0.002
Resp rate mean (median[IQR])	19.04 [16.67, 22.04]	19.50 [16.78, 22.80]	18.67 [16.56, 21.39]	<0.001	0.212	19.26 [16.81, 22.30]	19.46 [16.92, 22.44]	19.07 [16.73, 22.08]	0.088	0.065
SBP mean (median[IQR])	113.14 [104.64, 124.03]	112.52 [103.48, 124.55]	113.61 [105.79, 123.57]	0.006	0.045	113.54 [104.68, 124.87]	113.70 [104.08, 124.95]	113.43 [105.15, 124.78]	0.782	0.018
DBP mean (median[IQR])	59.76 [53.88, 66.47]	61.52 [55.54, 68.52]	57.87 [52.72, 64.24]	<0.001	0.348	59.41 [53.84, 66.00]	59.78 [54.00, 66.07]	58.96 [53.62, 65.70]	0.136	0.056
Urine output										
Urine output (median[IQR])	1,500.00 [895.00, 2,300.00]	1,500.00 [891.00, 2,380.00]	1,495.00 [900.00, 2,205.00]	0.186	0.075	1,421.00 [870.00, 2,181.25]	1,423.00 [893.25, 2,247.50]	1,420.50 [836.25, 2,155.00]	0.386	0.022
Severity score										
SOFA (median[IQR])	3.00 [2.00, 4.00]	3.00 [2.00, 4.00]	3.00 [2.00, 4.00]	0.024	0.059	3.00 [2.00, 4.00]	3.00 [2.00, 4.00]	3.00 [2.00, 4.00]	0.165	0.035
GCS (median[IQR])	14.00 [10.00, 14.00]	14.00 [10.00, 14.00]	14.00 [10.00, 14.00]	0.057	0.013	14.00 [10.00, 14.00]	14.00 [10.00, 14.00]	14.00 [11.00, 14.00]	0.848	0.011
APSIII (median[IQR])	49.00 [37.00, 66.00]	50.00 [37.00, 67.00]	48.00 [36.00, 65.00]	0.003	0.117	50.00 [38.00, 66.00]	49.00 [38.00, 65.75]	50.00 [38.00, 66.00]	0.924	0.007
OASIS (median[IQR])	35.00 [29.00, 41.00]	35.00 [29.00, 41.00]	35.00 [30.00, 41.00]	0.249	0.023	35.50 [29.00, 41.00]	35.50 [30.00, 41.00]	35.50 [29.00, 41.00]	0.962	0.014
Therapy										
Aspirin (%)										
No	2,252 (47.8)	1,718 (72.0)	534 (23.0)	<0.001	1.125	1,014 (45.3)	515 (46.1)	499 (44.6)	0.524	0.029
Yes	2,455 (52.2)	669 (28.0)	1,786 (77.0)			1,222 (54.7)	603 (53.9)	619 (55.4)		
Vasoactive agent (%)										
No	2,610 (55.4)	1,488 (62.3)	1,122 (48.4)	<0.001	0.284	1,286 (57.5)	647 (57.9)	639 (57.2)	0.765	0.014
Yes	2,097 (44.6)	899 (37.7)	1,198 (51.6)			950 (42.5)	471 (42.1)	479 (42.8)		
Ventilation (%)										
No	3,495 (74.3)	1,794 (75.2)	1,701 (73.3)	0.159	0.042	1,687 (75.4)	848 (75.8)	839 (75.0)	0.694	0.019
Yes	1,212 (25.7)	593 (24.8)	619 (26.7)			549 (24.6)	270 (24.2)	279 (25.0)		
Comorbidities										
Hypertension (%)										
No	2,798 (59.4)	1,544 (64.7)	1,254 (54.1)	<0.001	0.218	1,245 (55.7)	618 (55.3)	627 (56.1)	0.733	0.016
Yes	1,909 (40.6)	843 (35.3)	1,066 (45.9)			991 (44.3)	500 (44.7)	491 (43.9)		
Diabetes (%)										
No	3,422 (72.7)	1,962 (82.2)	1,460 (62.9)	<0.001	0.442	1,608 (71.9)	820 (73.3)	788 (70.5)	0.145	0.064
Yes	1,285 (27.3)	425 (17.8)	860 (37.1)			628 (28.1)	298 (26.7)	330 (29.5)		
Hyperlipidemia (%)										
No	4,395 (93.4)	2,213 (92.7)	2,182 (94.1)	0.073	0.054	2,098 (93.8)	1,048 (93.7)	1,050 (93.9)	0.930	0.007
Yes	312 (6.6)	174 (7.3)	138 (5.9)			138 (6.2)	70 (6.3)	68 (6.1)		
Cardiovascular disease (%)										
No	3,321 (70.6)	2,134 (89.4)	1,187 (51.2)	<0.001	0.921	1,740 (77.8)	870 (77.8)	870 (77.8)	1.000	<0.001
Yes	1,386 (29.4)	253 (10.6)	1,133 (48.8)			496 (22.2)	248 (22.2)	248 (22.2)		
PVD (%)										
No	4,489 (95.4)	2,326 (97.4)	2,163 (93.2)	<0.001	0.201	2,131 (95.3)	1,068 (95.5)	1,063 (95.1)	0.689	0.021
Yes	218 (4.6)	61 (2.6)	157 (6.8)			105 (4.7)	50 (4.5)	55 (4.9)		
Positive culture (%)										
No	3,542 (75.2)	1,756 (73.6)	1,786 (77.0)	0.007	0.079	1,650 (73.8)	821 (73.4)	829 (74.2)	0.736	0.016
Yes	1,165 (24.8)	631 (26.4)	534 (23.0)			586 (26.2)	297 (26.6)	289 (25.8)		
CCI (median[IQR])	5.00 [3.00, 7.00]	5.00 [2.00, 7.00]	6.00 [4.00, 8.00]	<0.001	0.440	6.00 [4.00, 8.00]	6.00 [4.00, 8.00]	6.00 [4.00, 7.00]	0.821	0.003

WBC, White Blood Cell; Bun, Blood Urea Nitrogen; PTT, Partial Thromboplastin Time; INR, International Normalized Ratio; SpO_2_, saturation of peripheral oxygen; Resp, respiration; SBP, systolic blood pressure; DBP, diastolic blood pressure; SOFA, Sepsis-related Organ Failure Assessment; GCS, Glasgow Coma Scale; APSIII, Acute Physiology Score III; OASIS, Oxford Acute Severity of Illness Score; PVD, Peripheral Vascular Disease; CCI, Charlson Comorbidity Index.

### Relationship between statin use and clinical outcomes

Table 2 summarized the comparison of clinical outcomes between the non-statin group and the statin group. According to Table 2, the mortality rates in the statin group were consistently lower than those in the non-statin group across various time points, including in-hospital, 30-day, 90-day, 180-day, and 365-day periods. The univariate analyses of cox proportional hazards model before PSM showed that statins could significantly reduce the mortality rate. The multivariate cox hazard analyses also demonstrated that patients in the statin group experienced a significantly lower in-hospital mortality rate (HR = 0.68, 95%CI [0.57–0.82], *p* < 0.001). Similar findings were observed for 30-day (HR = 0.69, 95%CI [0.59–0.82], *p* < 0.001), 90-day (HR = 0.76, 95%CI [0.66–0.86], *p* < 0.001), 180-day (HR = 0.77, 95%CI [0.68–0.87], *p* < 0.0001), and 365-day (HR = 0.78, 95%CI [0.69–0.87], *p* < 0.001) mortality rate. Interestingly, the beneficial effect of statins on reducing mortality remained consistently significant in three models after PSM, suggesting that the benefit of statins in reducing mortality was robust and consistent.

**Table 2:** Association between statin therapy and mortality of SAE patients.

Outcome	Before PSM						After PSM					
	Total (*n* = 4,707)	Non-statin (*n* = 2,387)	Statin (*n* = 2,320)	Model	HR [95% CI]	*p*	Total (*n* = 2,236)	Non-statin (*n* = 1,118)	Statin (*n* = 1,118)	Model	HR [95% CI]	*p*
In-hospital mortality, *n* (%)	683 (14.5)	412 (17.3)	271 (11.7)	1	0.71 [0.61–0.83]	<0.001	347 (15.5)	192 (17.2)	155 (13.9)	1	0.76 [0.61–0.94]	0.01
				2	0.55 [0.47–0.64]	<0.001				2	0.79 [0.64–0.97]	0.03
				3	0.68 [0.57–0.82]	<0.001				3	0.71 [0.57–0.89]	0.002
30-day mortality, *n* (%)	842 (17.9)	506 (21.2)	336 (14.5)	1	0.65 [0.57–0.75]	<0.001	428 (19.1)	244 (21.8)	184 (16.5)	1	0.73 [0.60–0.88]	0.001
				2	0.52 [0.45–0.60]	<0.001				2	0.76 [0.62–0.92]	0.004
				3	0.69 [0.59–0.82]	<0.001				3	0.68 [0.56–0.83]	<0.001
90-day mortality, *n* (%)	1,194 (25.4)	681 (28.5)	513 (22.1)	1	0.73 [0.65–0.82]	<0.001	612 (27.4)	334 (29.9)	278 (24.9)	1	0.79 [0.68–0.93]	0.004
				2	0.76 [0.66–0.86]	<0.001				2	0.83 [0.71–0.97]	0.02
				3	0.76 [0.66–0.86]	<0.001				3	0.77 [0.65–0.91]	0.002
180-day mortality, *n* (%)	1,401 (29.8)	787 (33.0)	614 (26.5)	1	0.75 [0.68–0.84]	<0.001	714 (31.9)	390 (34.9)	324 (29.0)	1	0.79 [0.68–0.91]	0.002
				2	0.59 [0.53–0.66]	<0.001				2	0.82 [0.71–0.95]	0.009
				3	0.77 [0.68–0.87]	<0.001				3	0.77 [0.66–0.90]	0.001
365-day mortality, *n* (%)	1,649 (35.0)	911 (38.2)	738 (31.8)	1	0.78 [0.71–0.86]	<0.001	849 (38.0)	462 (41.3)	387 (34.6)	1	0.79 [0.69–0.90]	<0.001
				2	0.61 [0.55–0.67]	<0.001				2	0.82 [0.72–0.94]	0.005
				3	0.78 [0.69–0.87]	<0.001				3	0.77 [0.67–0.88]	<0.001

SAE, sepsis-associated encephalopathy; PSM, propensity score matching; HR, Hazard Ratio; CI, Confidence Interval; WBC, White Blood Cell; Bun, Blood Urea Nitrogen; PTT, Partial Thromboplastin Time; INR, International Normalized Ratio; Resp, respiration; SBP, systolic blood pressure; DBP, diastolic blood pressure; SOFA, Sepsis-related Organ Failure Assessment; GCS, Glasgow Coma Scale; APSIII, Acute Physiology Score III; OASIS, Oxford Acute Severity of Illness Score; PVD, Peripheral Vascular Disease; CCI, Charlson Comorbidity Index.

Model 1: Unadjusted.

Model 2: Adjust for Age, Weight, Gender, Race.

Model 3: Adjust for Age, Weight, Gender, Race, Marital_status, Insurance, WBC, Platelet, Hemoglobin, Potassium, Sodium, Glucose, Creatinine, Bun, Aniongap, Bicarbonate, PTT, INR, SpO_2__mean, Heart_rate_mean, Temperature_mean, Resp_rate_mean, SBP_mean, DBP_mean, Urineoutput, SOFA, GCS, APSIII, OASIS, Aspirin, Vasoactive_agent, Ventilation, Hypertension, Diabetes, Hyperlipidemia, Cardiovascular_disease, PVD, Positive_culture, CCI.

**Figure 2:**
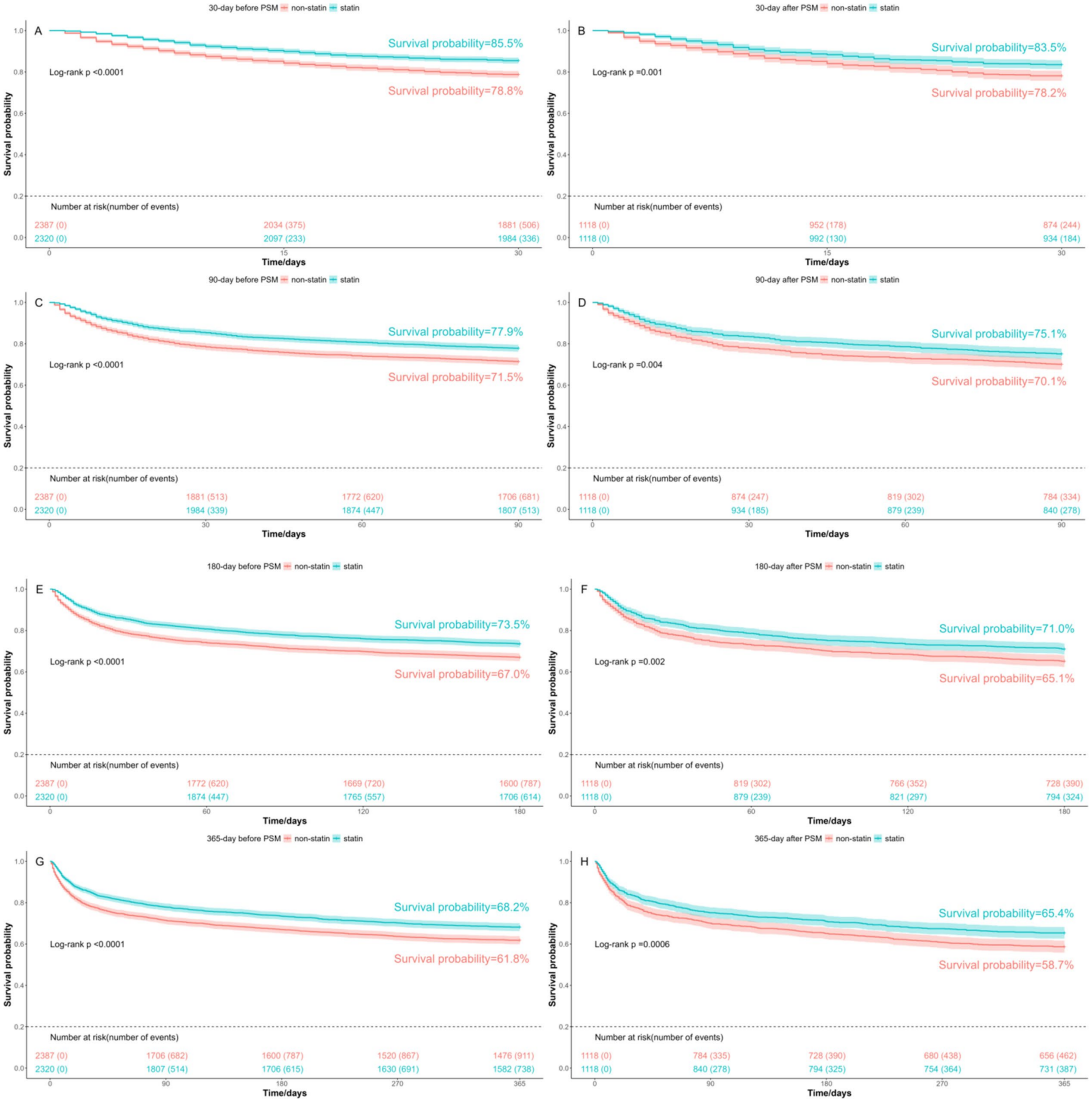
Kaplan-Meier survival curves between the non-statin and statin group. **(A)** 30-day mortality before PSM; **(B)** 30-day mortality after PSM; **(C)** 90-day mortality before PSM; **(D)** 90-day mortality after PSM; **(E)** 180-day mortality before PSM; **(F)** 180-day mortality after PSM; **(G)** 365-day mortality before PSM; **(H)** 365-day mortality after PSM. Group: non-statin group and statin group.

The Kaplan–Meier curves revealed that patients received statin therapy had improved survival probabilities compared to those who did not receive statins, both before PSM and after PSM. This trend persisted at 30-day, 90-day, 180-day, and 365-day, with all differences being statistically significant as indicated by a log-rank test *p*-value <0.05 (Figure 2).

### Subgroup analysis

**Figure 3:**
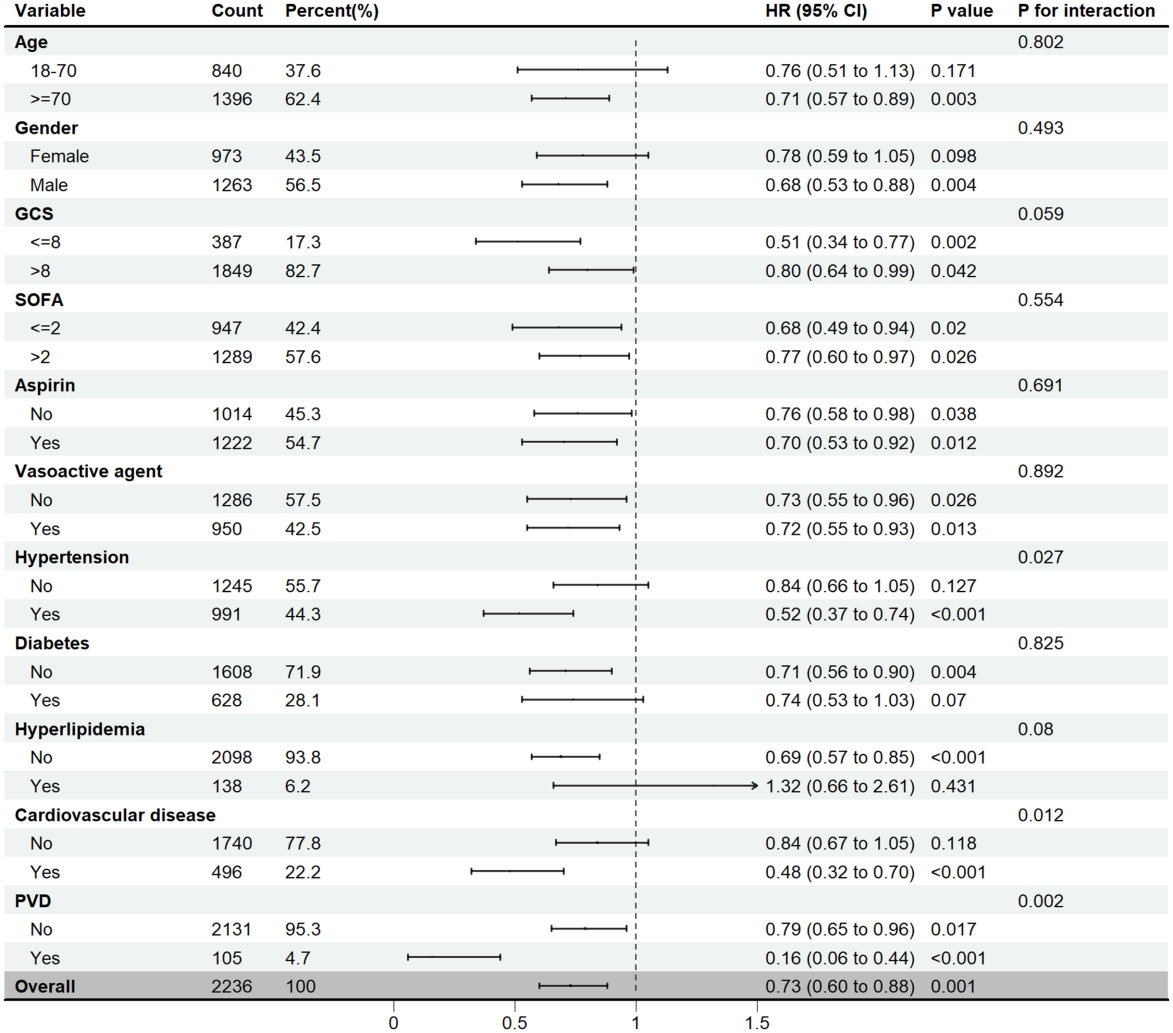
Subgroup analysis of the 30-day mortality in SAE patients, HR, hazard ratio; CI, confidence interval; GCS, Glasgow Coma Scale; SOFA, Sepsis-related Organ Failure Assessment; PVD, peripheral Vascular Disease.

A subgroup analysis was conducted based on the age, gender, GCS, SOFA scores, aspirin, vasoactive agent, hypertension, diabetes, hyperlipidemia, cardiovascular disease, and peripheral vascular disease (PVD). The findings indicated that statin therapy was associated with a significant 27% reduction in the 30-day mortality rate (HR = 0.73, 95%CI [0.60–0.88], *p* = 0.001). It seemed that statins were particularly effective in patients aged ≥70 years, male, and those with GCS score ≤ 8. Additionally, patients who received aspirin and vasoactive agents, as well as those comorbid with hypertension, cardiovascular disease, and PVD, experienced a more pronounced benefit from statin therapy. An interaction effect was observed in hypertension, cardiovascular disease, and PVD (Figure 3).

### Relationship between atorvastatin and clinical outcomes

**Figure 4:**
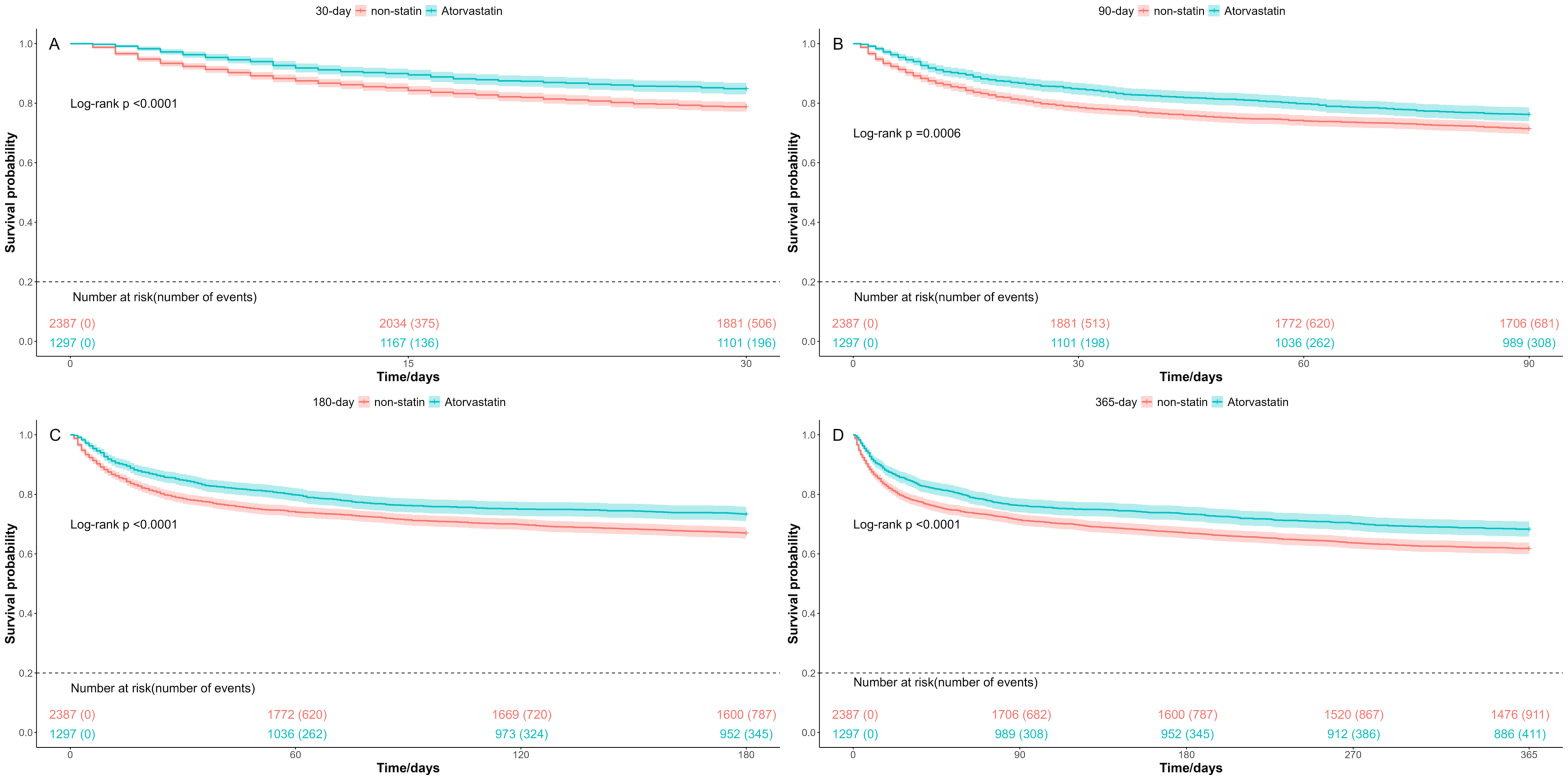
Kaplan-Meier survival curves for atrovastatin. **(A)** 30-day mortality; **(B)** 90-day mortality; **(C)** 180-day mortality; **(D)** 365-day mortality. Group: non-statin group and atrovastatin group.

In our study, atorvastatin emerged as the most commonly prescribed statin, with a total of 1,297 patients receiving it. Thus, we conducted a further analysis to explore the effect of atorvastatin on the prognosis of SAE patients. As showed in Table 3, the mortality rates among patients treated with atorvastatin were consistently lower compared to those in the non-statin group. Specifically, the in-hospital mortality was 17.3% in the non-statin group versus 13.0% in the atorvastatin group (HR = 0.73, 95%CI [0.59–0.91], *p* = 0.005). Similarly, the 30-day mortality was 21.2% versus 15.1% (HR = 0.72, 95%CI [0.59–0.87], *p* < 0.001), the 90-day mortality was 28.5% versus 23.7% (HR = 0.82, 95%CI [0.70–0.97], *p* = 0.017), the 180-day mortality was 33.0% versus 26.6% (HR = 0.79, 95%CI [0.68–0.91], *p* = 0.002), and the 365-day mortality was 38.2% versus 31.7% (HR = 0.79, 95% CI [0.68–0.90], *p* < 0.001). The Kaplan–Meier curves indicated that the survival probabilities of patients in the atorvastatin group were significantly higher than those in the non-atorvastatin group, for 30-day, 90-day, 180-day, and 365-day (Figure 4). It was observed that the effectiveness of atorvastatin treatment was similar to that of overall statin therapy.

**Table 3:** Association between atorvastatin and mortality of SAE patients.

Outcome	Total (*n* = 3,684)	Non (*n* = 2,387)	Atorvastatin (*n* = 1,297)	HR [95% CI]	*p*
In-hospital mortality, *n* (%)	580 (16.0)	412 (17.3)	168 (13.0)	0.73 [0.59–0.91]	0.005
30-day mortality, *n* (%)	702 (19.0)	506 (21.2)	196 (15.1)	0.72 [0.59–0.87]	<0.001
90-day mortality, *n* (%)	989 (27.0)	681 (28.5)	308 (23.7)	0.82 [0.70–0.97]	0.017
180-day mortality, *n* (%)	1,132 (31.0)	787 (33.0)	345 (26.6)	0.79 [0.68–0.91]	0.002
365-day mortality, *n* (%)	1,322 (36.0)	911 (38.2)	411 (31.7)	0.79 [0.68–0.90]	<0.001

### Relationship between statin dosage and clinical outcomes

**Figure 5:**
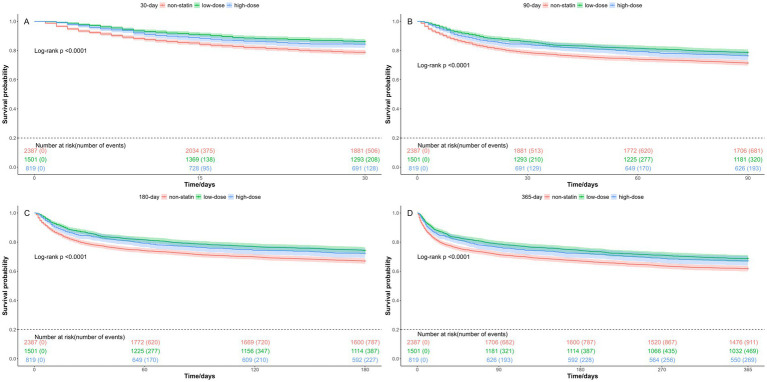
Kaplan-Meier survival curves of different statin dose group. **(A)** 30-day mortality; **(B)** 90-day mortality; **(C)** 180-day mortality; **(D)** 365-day mortality. Group: non-statin group, low-dose statin group, and high-dose statin group.

As to the statin dosage, high-dose statins was defined as atorvastatin 80 mg, simvastatin 80 mg, pravastatin 40 mg, and rosuvastatin 20 mg per day (9, 16). As illustrated in Table 4, both low-dose and high-dose statin groups demonstrated a significant reduction in mortality rates for in-hospital, 30-day, 90-day, 180-day, and 365-day. Kaplan–Meier survival analysis revealed that the survival probabilities for both groups were notably higher compared to the non-statin group, with low-dose group exhibiting the highest survival probability (Figure 5). All log-rank test *p*-values were < 0.05.

**Table 4:** Association between statin dose and mortality of SAE patients.

Outcome	Total (*n* = 4,707)	Non (*n* = 2,387)	Low-dose statin (*n* = 1,501)	HR [95% CI]	*p*	High-dose statin (*n* = 819)	HR [95% CI]	*p*
In-hospital mortality, *n* (%)	683 (15.0)	412 (17.3)	160 (10.7)	0.65 [0.53–0.80]	<0.001	111 (13.6)	0.74 [0.58–0.94]	0.01
30-day mortality, *n* (%)	842 (18.0)	506 (21.2)	208 (13.9)	0.67 [0.56–0.81]	<0.001	128 (15.6)	0.74 [0.59–0.92]	0.006
90-day mortality, *n* (%)	1,194 (25.0)	681 (28.5)	320 (21.3)	0.74 [0.63–0.85]	<0.001	193 (23.6)	0.80 [0.66–0.95]	0.01
180-day mortality, *n* (%)	1,401 (30.0)	787 (33.0)	387 (25.8)	0.75 [0.66–0.86]	<0.001	227 (27.7)	0.80 [0.68–0.95]	0.009
365-day mortality, *n* (%)	1,649 (35.0)	911 (38.2)	469 (31.2)	0.77 [0.68–0.87]	<0.001	269 (32.8)	0.81 [0.69–0.94]	0.006

### Relationship between statin exposure time and clinical outcomes

**Figure 6:**
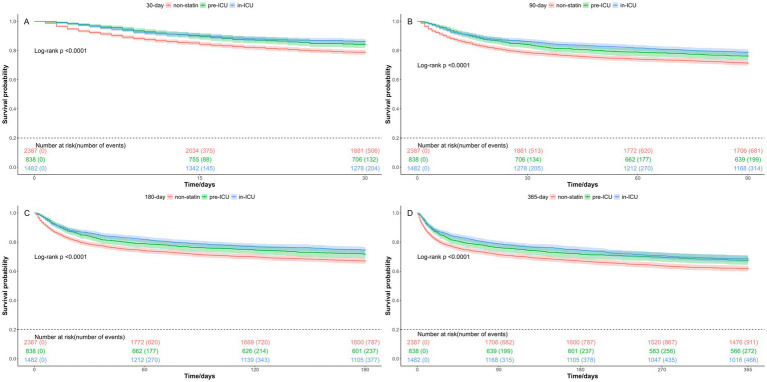
Kaplan-Meier survival curves for different statin exposure time. **(A)** 30-day mortality; **(B)** 90-day mortality; **(C)** 180-day mortality; **(D)** 365-day mortality. Group: non-statin group, pre-ICU statin group, and in-ICU statin group.

To investigate the optimal timing of statin administration, we categorized the patients into three groups: the non-statin group, the pre-ICU statin group, and the in-ICU statin group. The results showed that, compared to the non-statin group, both the pre-ICU and in-ICU statin groups experienced significantly lower in-hospital mortality rate, as well as reduced mortality rates at 30-day, 90-day, 180-day, and 365-day (Table 5). As presented in Figure 6 by Kaplan–Meier curves, the survival probabilities were markedly higher in both the pre-ICU group and in-ICU statin group than in the non-statin group, with the highest survival probability observed in the in-ICU statin group.

**Table 5:** Association between statin exposure time and mortality of SAE patients.

Outcome	Total (*n* = 4,707)	Non (*n* = 2,387)	Pre-ICU (*n* = 838)	HR [95% CI]	*p*	In-ICU (*n* = 1,482)	HR [95% CI]	*p*
In-hospital mortality, *n* (%)	683 (15.0)	412 (17.3)	113 (13.5)	0.74 [0.58–0.93]	0.009	158 (10.7)	0.64 [0.52–0.79]	<0.001
30-day mortality, *n* (%)	842 (18.0)	506 (21.2)	132 (15.8)	0.74 [0.60–0.91]	0.005	204 (13.8)	0.67 [0.56–0.80]	<0.001
90-day mortality, *n* (%)	1,194 (25.0)	681 (28.5)	199 (23.7)	0.80 [0.67–0.95]	0.01	314 (21.2)	0.73 [0.63–0.85]	<0.001
180-day mortality, *n* (%)	1,401 (30.0)	787 (33.0)	237 (28.3)	0.80 [0.68–0.94]	0.007	377 (25.4)	0.75 [0.65–0.86]	<0.001
365-day mortality, *n* (%)	1,649 (35.0)	911 (38.2)	272 (32.5)	0.77 [0.67–0.90]	<0.001	466 (31.4)	0.78 [0.69–0.89]	<0.001

## Discussion

Sepsis-associated encephalopathy (SAE) refers to septic patients with GCS score <15 or delirium. Given its high incidence and mortality rates, coupled with the absence of specific treatment intervention, there is an urgent need for further investigation into novel therapeutic approaches to improve patient outcomes.

Statins have been reported to be correlated with decreased mortality in sepsis and its associated complications. A real-world study demonstrated that the persistent use of various statins, including atorvastatin, rosuvastatin, pitavastatin, simvastatin, fluvastatin, and lovastatin, was associated with a significant reduction in the incidence of sepsis in patients with type 2 diabetes mellitus (T2DM). Moreover, statin users experienced a marked decrease in the incidence of septic shock compared to non-users (17). In patients with end-stage kidney disease who were hospitalized due to sepsis, statin use was associated with lower 30-day and 90-day mortality rates, both before and after the application of inverse probability treatment weighting (IPTW) (18). Mao et al. discovered that patients with acute respiratory distress syndrome (ARDS) who received statins pre-ICU admission exhibited a reduction in 30-day and 90-day mortality rates after adjusting for potential confounding factors (10). Yao et al. revealed the beneficial effect of statin therapy in reducing in-hospital mortality rate among patients suffering from sepsis-induced coagulopathy during ICU stay (19). Zhang et al. found that the combination of atorvastatins and imipenem suppressed the generation of neutrophil extracellular traps via the ERK/NOX2 signaling pathway, which significantly alleviated sepsis-induced lung injury, and improved the 7-day survival rate in septic mice (20). Yang et al. found that statin use among septic patients was associated with a lower incidence of acute pulmonary embolism (APE), with both standard and high doses of statins linked to a reduced risk of APE (21). Li et al. revealed that, the administration of statins could reduce 90-day and in-hospital mortality rates following ICU admission for sepsis, most likely attributable to its pleiotropic properties. However, the specific type of statins, the precise time of statins initiation, and the particular sepsis subpopulation were still unclear (22).

In this cohort study, we used data from the MIMIC database to investigate the association between statin administration and the risk of in-hospital mortality, as well as the risk of 30-day, 90-day, 180-day and 365-day mortality, among SAE patients admitted to the ICU. Our results demonstrated that statin use significantly reduced both short-term and long-term mortality risk in SAE patients. While previous research has described the association between statin and 30-day mortality in SAE patients, our study further demonstrates the protective effect of statins in this population-not only in the short term (30 days), but also in the extended periods of 90 days, 180 days, and 365 days. This finding significantly advances the insights of earlier studies (23). Further analysis of patients prescribed atorvastatin confirmed that the protective effect remained consistent, suggesting that the benefits of statins use in SAE were not dependent on the specific type of statin used. We also examined the impact of statin dosage on the prognosis of SAE patients and discovered that the protective effects persisted, regardless of whether patients received low-dose or high-dose statin therapy. Additionally, we investigated the optimal timing of statin administration and found that the protective effects sustained irrespective of whether the treatment was initiated pre-ICU or in-ICU admission. Taken together, these results emphasized that statin therapy was an independent protective factor for SAE, potentially offering clinicians a novel therapeutic approach for managing SAE. When treating SAE patients, doctors might consider adding statins into the treatment plan. If statins can reduce the mortality risks for these patients, their use could help alleviate the unnecessary suffering and prevent fatalities associated with this serious condition.

The association between statins and reduced mortality of SAE patients can be attributed to several factors. Firstly, statins could inhibit the generation of proinflammatory cytokines during sepsis, which help to alleviate systemic inflammation and prevent sepsis from progressing to a severe stage (24–27). Secondly, statins have been shown to exert antioxidant effects by modulating the nuclear factor erythroid 2 related factor 2/heme oxygenase-1 (Nrf2/HO-1) pathway (28). Thirdly, statins could regulate the immune responses by downregulating the synthesis of major histocompatibility complex-II, leading to decreased T cell proliferation and differentiation (29, 30). Finally, statins could activate AMPK and inhibit the RhoA/ROCK signaling pathway, which promoted eNOS activation, and then protect the vascular endothelium (29, 31).

In the subgroup analysis, we discovered that statin treatment had more favorable effects in male patients and those aged 70 years or older. This might be attributed to the pronounced inflammation in these individuals, allowing statins to exert their anti-inflammatory effects more effectively. Statins demonstrated notable protective effects in patients with GCS < 8, suggesting a greater benefit for those with more severe impairments in consciousness. Additionally, patients taking aspirin also exhibited greater benefits, indicating a potential synergistic effect between statins and aspirin (32). Statin therapy was more effective in individuals with hypertension, cardiovascular disease, and peripheral vascular disease (PVD), which might be related to the vasoprotective effects of statins. Additionally, it may be attributed to the shared pathophysiological features of atherosclerosis and sepsis, such as systemic inflammation, increased thrombosis, and immune dysregulation (32, 33). However, statin treatment appeared to be more effective in patients without hyperlipidemia. This result might be biased due to the limited number of patients with hyperlipidemia, and further study with a larger sample size was needed. Overall, the subgroup analysis proved valuable for customizing personalized treatment strategies for SAE patients, ensuring that statins were primarily prescribed to those most likely to experience their benefits.

The study had several limitations. Firstly, this study was a retrospective analysis based on a database, which made it impossible to control for potential confounding variables. Secondly, there was an amount of missing lipid data and inflammatory factors in the database, such as interleukin-6, C-reactive protein, and procalcitonin, preventing us from conducting statistical analysis on these indicators. Thirdly, we only demonstrated the association between statins and the prognosis of SAE patients, but we were unable to elucidate the specific mechanism of action of statins. In addition, we acknowledge that the observed associations may be mediated by immortal time bias, and the inability to perform target trial emulation. It is necessary to conduct large-scale prospective studies to further explore the relationship between statins and the prognosis of patients with SAE.

## Conclusion

The use of statins was associated with short-term and long-term mortality among SAE patients admitted to the ICU. This association was observed irrespective of statin type, dosage, or exposure time.

## Data Availability

The datasets presented in this study can be found in online repositories. The names of the repository/repositories and accession number(s) can be found in the article/Supplementary material.

## Glossary

SAE: sepsis-associated encephalopathy
ICU: intensive care unit
ARDS: acute respiratory distress syndrome
GCS: Glasgow coma scale
WBC: white blood cell
Bun: blood urea nitrogen
PTT: partial thromboplastin time
INR: international normalized ratio
SpO_2_: saturation of peripheral oxygen
Resp Rate Mean: respiratory rate mean
SBP: systolic blood pressure
DBP: diastolic blood pressure
SOFA: sepsis-related organ Failure Assessment
APSIII: acute physiology score III
OASIS: oxford acute severity of illness score
PVD: peripheral vascular disease
CCI: charlson comorbidity index
PSM: propensity score matching
HR: hazard ratio
CI: confidence interval
IQR: interquartile range
SMD: standardized mean differences
T2DM: type 2 diabetes mellitus
IPTW: inverse probability treatment weighting
APE: acute pulmonary embolism
