# Correction: Protective effect of statins in patients with sepsis-associated encephalopathy: a retrospective cohort study

**DOI:** 10.3389/fneur.2026.1860774

**Published:** 2026-05-05

**Authors:** Tieqiao Zhou, Lei Li, Jiping Yi, Xiaoxiang Gong, Fengzhen Huang

**Affiliations:** 1 The First People's Hospital of Chenzhou Affiliated to the University of South China, Chenzhou, Hunan, China; 2 The First Affiliated Hospital of Xiangnan University, Chenzhou, Hunan, China; 3 Department of Pediatrics, The Second Xiangya Hospital, Central South University, Changsha, Hunan, China

**Keywords:** MIMIC IV database, mortality, sepsis, sepsis-associated encephalopathy, statins

In the published article, the original 4 affiliations (below) included some errors:

^1^ Department of Laboratory Medicine, The First People's Hospital of Chenzhou Affiliated to the University of South China, Chenzhou, Hunan, China

^2^ Department of Neurology, The First People's Hospital of Chenzhou Affiliated to the University of South China, Chenzhou, Hunan, China

^3^ Department of Neurology, The First Affiliated Hospital of Xiangnan University, Chenzhou, Hunan, China

^4^ Department of Pediatrics, The Second Xiangya Hospital, Central South University, Changsha, Hunan, China

The updated affiliations appear below:

^1^ The First People's Hospital of Chenzhou Affiliated to the University of South China, Chenzhou, Hunan, China

^2^ The First Affiliated Hospital of Xiangnan University, Chenzhou, Hunan, China

^3^ Department of Pediatrics, The Second Xiangya Hospital, Central South University, Changsha, Hunan, China

The original version of this article has been updated.

